# Effect of Cycle-Based High-Intensity Interval Training and Moderate to Moderate-Intensity Continuous Training in Adolescent Soccer Players

**DOI:** 10.3390/healthcare9121628

**Published:** 2021-11-25

**Authors:** Bin Fang, Yonghwan Kim, Moonyoung Choi

**Affiliations:** 1 College of Physical Education, Luoyang Normal University, Luoyang 471934, China; fangbin@lynu.edu.cn; 2 Department of Physical Education, Gangneung-Wonju National University, Gangneung 25457, Korea; yhkim@gwnu.ac.kr; 3 Department of Sports Science Convergence, Dongguk University, Seoul 04620, Korea

**Keywords:** high-intensity interval training, moderate-intensity continuous training, anaerobic power, strength, cardiorespiratory fitness, soccer, adolescent, cycle-based training

## Abstract

Cardiorespiratory fitness, anaerobic power, and lower extremity strength are essential for soccer players at all levels. An effective program should be developed to improve physical strength for adolescent soccer players who need to combine academic and technical training. This study analyzed the impact of short-term high intensity interval training (HIIT) training and traditional moderate intensity continuous training (MICT) on adolescent soccer players. Participants included 56 adolescent soccer players who were divided into HIIT and MICT groups. The training program was conducted 3 times a week for 4 weeks using cycle ergometer. Each session included the same resistance training program, and the characteristics of HIIT and MICT were applied to improve cardiorespiratory fitness and anaerobic power. Body composition analysis, graded exercise test for peak oxygen uptake (VO_2_ peak), Wingate anaerobic power test, and isokinetic knee strength test were performed. VO_2_ peak was improved in HIIT and MICT, but anaerobic threshold and heart rate recovery significantly improved in the HIIT group. Wingate anaerobic peak power had increased significantly in sets 1, 2, and 3 in the HIIT group, but showed significant improvement only in set 1 in the MICT group. The isokinetic strength improved significantly in the HIIT group at 60°/s and in the MICT group at 240°/s. There was no significant change in body composition in either group. In conclusion, short-term HIIT administered to adolescent soccer players effectively improved cardiorespiratory fitness in HIIT and MICT groups. While HIIT increased anaerobic threshold and power, MICT effectively improved muscle endurance. Short-term intensive training can be considered a time-efficient training strategy.

## 1. Introduction

Soccer players require physiological and physical conditioning to achieve optimal performance, depends on many factors, including technical, tactical, physical, physiological, and mental condition [1,2].

For optimal energy metabolism, soccer requires high aerobic capacity because the game lasts for 90 min. Moreover, since high-intensity activities that depend on anaerobic energy metabolism are restored using aerobic energy metabolism, active recovery through aerobic activity performed at a lower level than the lactate threshold is required after high-intensity short sprints [3]. The moderate intensity continuous training (MICT) is the traditional, most widely used training method. This method involves running for a long time at the level of about 55–75% of VO_2_ peak. A previous study suggested that a 12-week MICT running program improved VO_2_ peak by 21.5% [4,5].

Meanwhile, because soccer involves repeated intermittent high-intensity running, it is necessary to optimize the physiologically high-level anaerobic energy system [6]. The activities that have a decisive influence on the outcome of a soccer match include sprints, jumping, duels, and kicking, all of which require explosive power [7]. The average exercise intensity, measured by the maximum heart rate during the 90-min game, is reportedly close to the lactate threshold or anaerobic threshold level [2]. Soccer players spend 11% of their total competition time in high-intensity anaerobic activity, causing lactate accumulation, and run a high-intensity sprint every 90 s. Therefore, anaerobic capacity is a factor that significantly affects performance [8].

An increase in maximal strength generally leads to an increase in relative strength and muscle power. Previous studies have reported a significant proportional relationship of maximal strength with acceleration and movement speed [9]. Additionally, in soccer, the muscle contractility of the lower extremities can affect the improvement of very important skills in soccer matches, such as changing direction, sprinting, and changing speed [10]. The key factors for a successful soccer match are, therefore, related to maximal strength of the lower extremities and anaerobic power [7].

The high-intensity interval training (HIIT) can not only improve anaerobic capacity but also cardiorespiratory fitness by meeting these needs. HIIT involves repeating 5–45 s of high-intensity training and 2–4 min of recovery for up to 40 min [11]. Because HIIT is similar to soccer games, the intensity of training interval consists of over 80% with VO_2_ peak higher than the anaerobic threshold, and intensity of recovery interval is around 50% [5,12].

The HIIT is characterized by the ability to improve both aerobic and anaerobic metabolism and has been reported to provide more effective improvements than MICT [13]. After 8 weeks of HIIT training, the maximum oxygen uptake reportedly improved by +8%, the glycogen content acting as energy for the anaerobic system increased by +3%, and the recovery heart rate changed by −7% [14]. Also, studies conducted on youth soccer players have shown positive results. McMillan et al. [15], reported that VO2 max, skirt jump, and counter movement jump height were significantly improved after 10 weeks of HIIT. Moreover, Mosey’s study showed an improvement in time reduction of 3.5% as measured by the Intermittent Fitness Test with HIIT once a week for 8 weeks [15].

However, despite the positive effects of HIIT, adolescents with low physical fitness and low training compliance are more likely to drop out because they repeat and continue high-intensity training. A previous study on the prevalence and predictors of HIIT dropout reported a 17.6% dropout rate, and that long training periods, and long exercise session times [16]. However, HIIT performed on a stationary bicycle showed a significantly lower dropout rate than a form of training such as running [17]. Compared to plyometric or sprint training, HIIT using a stationary bicycle has the advantage of being able to monitor the heart rate response in real time and it is easy to observe the training effect according to the absolute training intensity [18]. Moreover, training using a stationary bicycle can be useful for soccer players even to prevent occupational overuse syndrome [19].

However, HIIT studies have mainly focused on running-based training, and there are not many studies examining whether short-term bicycle-based HIIT is effective for youth soccer players. Therefore, this study compared two groups of adolescent soccer players, one performing MICT and another performing HIIT using a bicycle ergometer, and analyzed changes in cardiorespiratory fitness, anaerobic power, fatigue index, isokinetic knee strength, and body composition.

## 2. Method

### 2.1. Participants

The participants were 83 boys (15–18 years old) high school soccer players who visited the player training center after seeing an announcement on the bulletin board. In consultation with the coach in charge, they chose between HIIT and MICT, and participated in the training for 4 weeks. By the end of the sessions, 13 patients undergoing rehabilitation due to knee, ankle, and back injuries since the beginning and 14 dropouts during the study were excluded from the analysis. Therefore, the final analysis included 27 participants in the HIIT group and 29 in the MICT group. Written informed consent from the participants as well as their guardians or team coaches was obtained. Approval from the Gangeung-Wonju National University Research Ethics Committee was obtained for this study (GWNUIRB-2021-11; approval date: 25 February 2021).

### 2.2. Body Composition

Body composition was measured using Inbody 720 (Inbody Co., Seoul, Korea), which uses bioelectrical impedance to measure the impedance of each part of the body using an 8-point tactile electrode system [20]. The Inbody device sends varying frequencies of alternating current to the body through hands and feet. The analyzer uses a tetrapolar 8-point tactile electrode system and performs 30 impedance measurements at six frequencies (1, 5, 50, 250, 500, 1000 kHz) with a test duration of about 60 s [21]. This device passes current through the body through resistors and capacitors, resistors measure extracellular and intracellular fluids, and capacitors measure cell membranes [22]. These impedance values are then used to predict several variables, including body fat percentage, fat mass, fat-free mass, and total body water. Compared to DXA, InBody devices tend to underestimate body fat percent and fat mass and overestimate fat-free mass but have the advantage that individual errors are relatively small [23]. Fat mass and muscle mass were measured as weight in kg. Fat and muscle ratios were converted into percentages by dividing the measured fat mass and muscle mass by body weight, respectively. For accurate measurement, the participants removed conductive metals and jewelry that came into contact with the skin. After wiping the hands and soles of their feet with an alcohol swab, they stood on the electrode scaffold. Measurements were performed in a position in which both hands were widened by about 30° after holding the electrode handle. To minimize measurement error, the intake of food, beverage, alcohol, caffeine, etc., as well as vigorous physical activity, were restricted for 8 h before measurement.

### 2.3. Cardiorespiratory Fitness

The peak oxygen uptake (VO_2_ peak) was measured for cardiorespiratory fitness (CRF). Additionally, anaerobic threshold and heart rate recovery were measured using a treadmill and a gas analyzer (Vmax229, Sensormedics Co., Yorba Linda, CA, USA). The test protocol used BURCE, and the intensity was gradually increased every 3 min [24]. Heart rates were measured at 1 min and 3 min after the test ended to measure the heart rate recovery rate. For the anaerobic threshold, the ventilation volume, VO_2_, and VCO_2_ recorded results were analyzed every 10 s after the end of the test. These three volumes gradually increased, and then the point showed a sudden increase [25].

The contents and purpose of the test were explained to the participant and guardian or coach, and a written consent form for the test was completed prior to proceeding. To prepare for the risk to the heart that may occur during the test and to ensure safety, an electrocardiogram analyzer (Case8000, GE Marquette Co., Cleveland, OH, USA) was utilized to continuously monitor and measure heart activity by a cardiologist. Blood pressure was checked every minute during the test, and the measurement value 1 min before the stage change and rating of perceived exertion (RPE) were measured every 3 min. Although the test was conducted on the premise of measuring the maximum CRF, the test was stopped if an abnormality was found on the electrocardiogram, the RPE was 17 or higher, the heart rate and oxygen intake did not increase despite the increase in exercise intensity, or the participant requested discontinuation based on their subjective symptoms [20]. Participants who did not reach 90% or more of the predicted maximum heart rate (220 − age) or prematurely terminated training were excluded from the analysis.

### 2.4. Anaerobic Power and Fatigue

Anaerobic power and fatigue were measured using the Wingate test. The Wingate test is the most commonly performed test to measure anaerobic capacity in athletes. The test consists of a 30-s sprint for a constant load applied according to body weight (0.075 kg^−1^ body mass) [26]. A friction-loaded cycle ergometer (Monark model 864 Crescent AB, Varberg, Sweden) was used for the measurement. The height of the saddle and handlebar was individually adjusted to fit the body structure of the participant. The saddle height was adjusted such that when the participant sat on the saddle and extended one leg as much as possible, it was at a 25- to 35-degree angle. First, for warm-up, the number of revolutions per minute was set to 80 revolutions per minute (RPM) under a light load of 1 kp (50 watts), and cycling was performed for 4 min. After a warm-up, the examiner counted down 5 s before the start and applied the calculated load simultaneously as the examiner’s “start” signal. The participant tried to exert maximal RPM for 30 s, and the examiner verbally encouraged the participant to continue maximal effort throughout the test. The inspector recorded the RPM every 5 s during the test, and after the test was finished, the Peak RPM, the lowest RPM, and the average RPM were recorded. Peak Power was calculated using the measured record.

Peak power = [(Peak RPM/12) × kp × (6/0.0833)]

The peak power measured as an absolute value was defined as the relative peak power using the value divided by the body weight. Fatigue index refers to the percentage of power lost from the start to the end of the test and was calculated by multiplying the difference between the peak RPM and the lowest RPM by the peak RPM by 100. A total of 3 sets were repeated for the test, and the rest period between sets was set to 2 min.

Fatigue index = [(peak RPM − minimum RPM)/peak RPM] × 100

### 2.5. Isokinetic Knee Strength

The isokinetic knee strength was measured using an isokinetic dynamometer (Humac Norm, CSMi, Stoughton, MA, USA) in extension and flexion strength of the knee joint. The isokinetic dynamometer is a computerized and mechanically speed-resisting force measuring device [27]. Angular velocities of 60, 180, and 240°/s were performed. Measurement for muscle strength was performed at an angular velocity of 60°/s, at an angular velocity of 180°/s for muscle power, and an angular velocity of 240°/s for muscle endurance.

Participants sat in a chair and aligned the dynamometer axis with the lateral epicondyle of the femur to align the knee anatomical axis. Measurements were made with uniaxial contractions for extension and flexion of the knee. The test method was explained to the participants, and several practice exercises were conducted to ensure understanding. The range of flexion and extension of the knee for the examination was set to 0–90 degrees, and the maximum knee-stretched position was set to 0 degrees. The knee was flexed 90 degrees. First, the examiner signaled to wait. The first extension measurement was made with the start signal, and flexion was measured with the next signal. The participant performed 4 measurements at an angular velocity of 60°/s, 4 times at an angular velocity of 180°/s, and 25 measurements at an angular velocity of near 240°/s. For muscle strength, peak torque was measured, and the unit was Newton meter (Nm). Muscle power was measured by average power using watts, and muscle endurance was measured by total work using joules. The measured absolute values of muscle strength, power, and endurance were calculated by dividing the value by body weight as a percentage, and the relative values of extensors and flexors were added for analysis.

### 2.6. Training Program

#### 2.6.1. High-Intensity Interval Training

The Repeated Wingate sprint protocol, as described within the HIIT program used by Breenfeldt et al. [28], was applied and performed 3 times a week for 4 weeks. The Repeated Wingate sprint protocol is a sprint interval training (SIT) method that involves an effort of 30 s close to maximum and “all out” Participants performed warm-up at 60 RPM for 10 min under a load of 1 kp corresponding to 50 W. After a warm-up, the load on the pedal was removed, and the participant performed unloaded pedaling, gradually increasing the pedaling speed and then starting this training immediately after reaching 100 RPM or more. Individual load (0.075 kg^−1^ body mass) according to their body weight was applied along with a start signal to the participants, and they were verbally encouraged to pedal as quickly as possible for 30 s. After 30 s of Wingate sprint, participants took an active rest while holding 60 RPM for 2 min at a load of 1 kp. During the last 5 s of the rest period, the participant again reached over 100 RPM, and the 30-s Wingate sprint was repeated. During the entire training, participants performed 6 sets of 30-s Wingate sprints and recovery 2-min intervals. After this exercise, cycling in break interval was performed at 60 RPM at 1 kp load for 10 min. Next, 6 sets of 30-s. Wingate sprints were once again repeated.

#### 2.6.2. Moderate-Intensity Continuous Training

The MICT program was conducted 3 times a week for 4 weeks in the same way as the HIIT group using the training program described in Ko et al. [18]. The MICT group performed continuous cycling for 60 min at moderate intensity, eliciting 55–75% of the VO_2_ peak using an electromagnetically braked cycle ergometer. To set the training intensity, the heart rate at 55–75% of VO_2_ peak was applied using the data output from the CRF test. An electronic heart rate monitoring device (Polar H10, Polar Electro, Bethpage, NY, USA) was used so that athletes and trainers could check in real-time.

#### 2.6.3. Resistance Training

HIIT and MICT groups completed the same resistance training (RT) programs. All training consisted of 12 sessions (3 times a week for 4 weeks). The RT program consisted of weight training focused on strengthening the muscles. Following the American College of Sports Medicine (ACSM) recommendations, 12 repetitions of 3 sets at 80% of 1 RM were performed to increase strength [29]. One repetition maximum (1 RM) measurement was performed on the first day, and the direct measurement method was performed by the researchers. Resistance exercise was applied equally to both groups. Participants performed resistance exercise after HIIT or MICT training was completed and after sufficient rest for 30 min or more. Weight training equipment included leg extension, leg curl, leg press, hip abduction, inner thigh, shoulder press, chest press, butterfly, lat pull-down, long pull, arm curl, and abdominal machines. The participants decided on the order in which they would use the equipment and move from one machine to another after their training.

### 2.7. Data Statistics

The G*power software (G*power 3.1, University of Düsseldorf, Düsseldorf, Germany) was used for sample size calculation. The conditions were as follows; Wilcoxon signed-rank test (one sample case), effect size d = 0.5; α error prob = 0.05; Power (1-β error probability) = 0.90. Data were analyzed using IBM SPSS Statistics for Windows/Macintosh, version 25.0 (IBM Corp., Armonk, NY, USA), and continuous variables were recorded as mean and standard deviation. As a result of performing the normality test with Kolmogorov–Smirnov and Shapiro–Wilk, the main analysis variables did not show a normal distribution; therefore, a nonparametric analysis was performed. The Wilcoxon method was used to perform intra-group of pre- and post-comparison. For comparison between groups, the Mann–Whitney method was used. The significance level was set at *p* < 0.05.

## 3. Results

### 3.1. General Characteristics

The participants were classified according to groups, and the general characteristics of the participants are shown in Table 1. When comparing the HIIT and the MICT groups, there was no statistically significant difference in age, height, weight, and body mass index (BMI).

### 3.2. Cardiorespiratory Fitness

In the change of VO_2_ peak before and after training, both the HIIT group and the MICT group showed a statistically significant increase after training (*p* = 0.012 and *p* = 0.028), while the anaerobic threshold showed a statistically significant increase after training only in the HIIT group (*p* = 0.025). In the heart rate recovery rate 1 min (*p* = 0.029) and 3 min (*p* = 0.015) after the end of the test, the recovery rate increased significantly after training only in the HIIT group (Table 2).

### 3.3. Wingate Test; Anaerobic Power and Fatigue Index

As for the change in anaerobic power, in set 1, both groups showed a statistically significant increase in peak power (*p* < 0.05). However, in sets 2 and 3, peak power increased significantly only in the HIIT group (*p* = 0.011 and *p* = 0.005). After training, in the HIIT group, there was no significant difference in set 2 compared with set 1, and the peak power decreased significantly in set 3 in the MICT group. However, set 2 and set 3 showed a significant decrease in terms of peak power compared to set 1 (Table 3).

Changes in fatigue index before and after training showed similar results to anaerobic peak power.

In the HIIT group, there was a significant improvement in sets 1, 2, and 3. However, in the MICT group, only set 1 showed a significant improvement, with no significant change in sets 2 and 3 (Table 4).

### 3.4. Isokinetic Knee Muscle Strength, Power, and Endurance

In terms of change in isokinetic knee muscle strength before and after training, at an angular velocity of 60°/s, the muscle strength increased significantly after training for both the HIIT and the MICT groups (*p* = 0.024 and *p* = 0.016). However, in comparison between groups, the HIIT group after training was significantly higher (*p* = 0.032). At an angular velocity of 180°/s, muscle power significantly increased in the HIIT group (*p* = 0.039), but at an angular velocity of 240°/s, muscle endurance significantly increased in the MICT group (*p* = 0.033) (Table 5).

### 3.5. Body Composition

Comparing the difference in body composition before and after training showed no statistically significant difference in total fat mass, fat ratio, muscle mass, and muscle ratio for body composition. In particular, after training, fat mass decreased in both groups, but the decrease was not significant (Table 6).

## 4. Discussion

Soccer is a sport that requires both aerobic and anaerobic capacity, and in this study, HIIT and MICT cycle ergometers were used to train youth soccer players for 4 weeks. Cardiorespiratory fitness and muscle strength improved in HIIT and MICT groups. While HIIT increased anaerobic threshold and power, and MICT significantly improved muscle endurance.

In a previous study on CRF, MacInnis et al. [30] reported that 6 sessions of short-term HIIT over 2 weeks elicited greater increases in citrate synthase maximal activity and mitochondrial respiration in skeletal muscle compared to MICT during total work and session duration. Through a meta-analysis, Cao et al. [31] confirmed that HIIT is a more effective training method for improving CRF in children and adolescents aged 6–17 years than MICT. In addition, various studies have shown that HIIT is more effective in the central adaptation of maximal stroke volume, cardiac output, and blood volume, which are major components of CRF [32,33]. The current study results suggested that anaerobic threshold and heart rate recovery improved significantly after training only in the HIIT group. In terms of VO_2_ peak, both the HITT and MICT groups improved significantly, but the HIIT group improved more after training. These results suggest that short-term HIIT may be a more effective training method for improving CRF than MICT, even for adolescent soccer players.

Previous studies have shown that various forms of HIIT have a significant effect on anaerobic power in athletes trained for endurance enhancement [34,35]. Stöggl et al. [35] reported that HIIT induces greater glycolytic activity, including the formation of lactic acid, and this activity favorably acts to generate ATP at a higher rate. Therefore, short-term HIIT is thought to affect both maximal anaerobic power and neuromuscular components significantly. In this study’s anaerobic power results, the peak power was significantly improved in both the HIIT and MICT groups in set 1, but only in the HIIT group in sets 2 and 3. In addition, the HIIT group maintained the peak power from set 1 to set 2 without a significant decrease. However, the MICT group showed a significant decrease in the peak power after set 1.

When measuring anaerobic fatigue in the Wingate test, the fatigue index is usually applied as a percentage of the data point’s power drop to peak power and lowest power. Therefore, a high value means high fatigue, which is harmful for performance [36]. In the results for fatigue in this study, the fatigue index was significantly improved only in the HIIT group in all 3 sets. In the HIIT group, fatigue did not increase significantly from set 1 to set 2, but the MICT group showed a significant increase in fatigue starting in set 2. These results suggest that short-term HIIT conducted using a cycle ergometer not only increased anaerobic power but also effectively strengthened endurance for repeated high-intensity anaerobic activities.

In soccer players, quadriceps play an important role in jumping and ball kicking, and hamstrings are known to control running activity and stabilize the knee joint during turns or tackles [37]. Also, the role of the quadriceps and hamstrings in joint stability becomes more important as speed increases [7]. The measurement of isokinetic knee strength in this study revealed that peak torque of 60°/s angular velocity, average power of 180°/s angular velocity, and total work of 240°/s angular velocity improved significantly in both groups. However, in the comparison between groups, the peak torque and average power were significantly higher in the HIIT group after training. In contrast, the total work in the MICT group became significantly stronger. Tabata et al. [38] reported a significant increase in the slow angular velocity tests of 30, 60, and 120°/s in participants who performed high-intensity endurance training at 90% VO_2_ peak using a cycle ergometer, but for 30, 60 s. This is consistent with our study results, which showed no significant difference in the fast angular velocity test of 120°/s. This suggests that HIIT may be more effective when the goal is to enhance muscle and muscle power, and MICT may be a more effective training strategy when the goal is to enhance muscle endurance. Previous studies found that HIIT did not increase the total muscle cross-sectional area but did increase the cross-sectional area ratio of certain type II muscle fibers [38]. In addition, HIIT is known to improve neuromuscular coordination, affect muscle strength, and increase muscle power [39]. Therefore, it is essential to adjust the ratio of HIIT and MICT in training programs according to their purpose rather than just performing HIIT when the goal is to improve muscle function.

Body composition is determined by the quantity and quality of several factors that affect an athlete’s performance and health [40]. When evaluating an athlete’s body composition, there is a report that the most meaningful results can be derived when quantitative and qualitative analysis are evaluated simultaneously [41]. Quantitative analysis can evaluate body mass and fat-free mass through total body, intracellular, and extracellular water content, and qualitative analysis can monitor intra/extracellular water ratio through R–Xc graph [42]. However, this study focused on the changes in CRF, anaerobic power, and muscle strength related to the athlete’s performance, and only quantitative analysis was performed to confirm the changes in muscle mass and body fat through training. The quantitative analysis of the athlete’s body composition suggested that there was no significant difference in all variables. In both HIIT and MICT, a period of 4 weeks is insufficient to affect body composition such as muscle and body fat. Previous studies have questioned whether HIIT has a greater effect on improving body composition than MICT [43,44]. Smith et al. [43] reported that there was no significant effect difference in body composition change between HIIT and MICT for 3 weeks; Nybo et al. [44] also reported that short-term HIIT was effective for improving CRF but had no significant effect on muscle mass increase and body fat loss compared to long-term training. Short-term HIIT may be an effective training method in terms of time efficiency. However, in this study, it did not affect body composition. Therefore, when the goal is to improve body composition, both HIIT and MICT should be implemented with long-term goals and plans rather than short-term training.

The limitation of this study is that while soccer is a sport based on running, training is based on bicycles. Also, since only men were included in the study, the effects of bicycle HIIT and MICT cannot be generalized to women. In addition, since it was a short-term training, additional research should be conducted on the effect of continuous training. In our current study, a choice between the two training types was not assigned randomly. Instead, after discussing with the players and coaching staff, their preferred method was assigned. In particular, because players have to train at high intensity, the researchers adopted a method to comply with ethical guidelines.

In this study, both HIIT and MICT improved VO_2_ max. According to the previous study, these changes were non-linear in the two groups, and there was a difference between groups [45]. Specifically, it was reported that the continuous training group showed changes in cardiorespiratory variables before and after the lactate threshold, and that HIIT showed changes in physiological variables after the lactate threshold [46]. Based on this information, future research should be conducted to find the factors of change in the cell unit, muscle, and cardiopulmonary system according to the training method and intensity.

## 5. Conclusions

After four weeks of short-term training, VO_2_ peak, anaerobic threshold, heart rate recovery, anaerobic power, fatigue index, and isokinetic knee muscle strength and power significantly improved in the HIIT group. In the MICT group, VO_2_ peak, muscle strength, and muscular endurance showed improvement. These findings suggest that, even for a short period of time, HIIT can help achieve improvement in the aerobic as well as anaerobic power, and recovery capacity that affect the performance improvement of adolescent soccer players compared to MICT. Therefore, short-term HIIT application is considered to be a time-efficient training strategy for adolescent soccer players who need to combine education and training.

## Figures and Tables

**Table 1:** General Characteristics.

Variables	HIIT (*n* = 27)	MICT (*n* = 29)	*t*	*p*-Values
Age, years	15.7 ± 0.8	15.8 ± 0.7	−0.506	0.615
Height, cm	176.0 ± 5.5	178.7 ± 5.3	−1.884	0.064
Weight, kg	64.3 ± 6.6	65.8 ± 6.3	−0.891	0.377
BMI, kg/m^2^	20.8 ± 2.0	20.6 ± 1.5	0.383	0.703

Abbreviations: HIIT, high-intensity interval training; MICT, moderate-intensity continuous training; BMI, body mass index.

**Table 2:** Exercise Graded Test with Cardiorespiratory Fitness.

Variables	Group	Pre	Post	Difference (%)	*p*-Values
VO_2_ peak, mL/kg/min	HIIT	47.7 ± 6.7	55.3 ± 7.5	15.9	0.012 *
	MICT	48.1 ± 7.4	52.2 ± 8.9	8.5	0.028 *
	*p*-values	0.524	0.041 *		
Anaerobic Threshold, %	HIIT	64.2 ± 8.9	73.5 ± 6.6	16.6	0.025 *
	MICT	65.4 ± 7.3	68.0 ± 6.9	4.0	0.138
	*p*-values	0.495	0.031 *		
Recovery 1 m HR, %	HIIT	55.7 ± 6.8	70.0 ± 11.8	25.7	0.029 *
	MICT	56.2 ± 7.4	61.6 ± 12.0	9.6	0.217
	*p*-values	0.413	0.013 *		
Recovery 3 m HR, %	HIIT	82.2 ± 13.8	92.0 ± 14.5	11.9	0.015 *
	MICT	80.4 ± 17.2	85.6 ± 16.3	6.5	0.188
	*p*-values	0.554	0.038 *		

* *p* < 0.05; Abbreviations: HIIT, high-intensity interval training; MICT, moderate-intensity continuous training; HR, heart rate.

**Table 3:** Peak Power per Body Weight with Wingate Test, (Peak Power/kg).

Variables	Set	Pre	Post	Difference (%)	*p*-Values
HIIT	1	11.2 ± 2.2	12.6 ± 1.3	12.5	0.016 *
	2	10.3 ± 1.4 ^a^	11.9 ± 1.6	15.5	0.011 *
	3	9.7 ± 1.6 ^b,c^	10.8 ± 1.7 ^c^	11.3	0.005 *
	*p*-values	0.034 *	0.022 *		
MICT	1	11.7 ± 1.2	12.5 ± 1.1	6.8	0.040 *
	2	10.2 ± 1.3 ^a^	10.8 ± 1.5 ^a^	5.9	0.254
	3	9.4 ± 1.5 ^b,c^	9.6 ± 1.2 ^b,c^	2.1	0.163
	*p*-values	0.026 *	0.022 *		

* *p* < 0.05; ^a^, 1 set versus 2 set; ^b^, 2 set versus 3 set; ^c^, 1 set versus 3 set. Abbreviations: HIIT, high-intensity interval training; MICT, moderate-intensity continuous training.

**Table 4:** Fatigue Index with Wingate Test.

Variables	Set	Pre	Post	Difference (%)	*p*-Values
HIIT	1	41.0 ± 12.2	35.3 ± 7.7	−14.6	0.023 *
	2	47.9 ± 10.4 ^a^	42.5 ± 9.8	−11.3	0.046 *
	3	59.4 ± 9.4 ^b,c^	48.7 ± 10.7 ^c^	−18.0	0.016 *
	*p*-values	0.013 *	0.040 *		
MICT	1	43.6 ± 11.9	37.7 ± 9.2	−13.8	0.032 *
	2	49.5 ± 10.2 ^a^	46.9 ± 10.6 ^a^	−5.3	0.315
	3	57.0 ± 11.5 ^b,c^	55.9 ± 13.1 ^b,c^	−1.9	0.265
	*p*-values	0.028 *	0.019 *		

* *p* < 0.05; ^a^, 1 set versus 2 set; ^b^, 2 set versus 3 set; ^c^, 1 set versus 3 set. Abbreviations: HIIT, high-intensity interval training; MICT, moderate-intensity continuous training.

**Table 5:** Isokinetic Knee Strength Test.

Variables	Group	Pre	Post	Difference (%)	*p*-Values
60°/s, Nm/kg, %	HIIT	487.8 ± 127.1	532.5 ± 120.4	9.2	0.024 *
	MICT	477.6 ± 116.0	506.8 ± 107.8	6.0	0.016 *
	*p*-values	0.724	0.032 *		
180°/s, Watt/kg, %	HIIT	618.3 ± 127.0	649.8 ± 135.7	5.0	0.039 *
	MICT	607.6 ± 139.1	632.1 ± 120.2	4.1	0.231
	*p*-values	0.629	0.020 *		
240°/s, total Joule/kg	HIIT	60.6 ± 13.6	65.9 ± 15.1	8.7	0.140
	MICT	61.4 ± 12.3	70.5 ± 14.6	14.8	0.033 *
	*p*-values	0.431	0.039 *		

* *p* < 0.05; Abbreviations: HIIT, high-intensity interval training; MICT, moderate-intensity continuous training.

**Table 6:** Changes in Body Composition.

Variables	Group	Pre	Post	Difference (%)	*p*-Values
Fat mass, kg	HIIT	7.4 ± 1.9	7.1 ± 2.0	−4.1	0.174
	MICT	7.3 ± 2.1	7.0 ± 1.8	−4.1	0.141
	*p*-values	0.766	0.755		
Fat ratio, %	HIIT	11.4 ± 2.4	11.3 ± 1.9	−1.3	0.183
	MICT	11.2 ± 2.1	10.8 ± 2.2	−0.9	0.210
	*p*-values	0.576	0.481		
Muscle mass, kg	HIIT	31.0 ± 3.2	32.1 ± 3.0	3.5	0.774
	MICT	31.9 ± 3.1	32.2 ± 2.9	0.9	0.813
	*p*-values	0.499	0.425		
Muscle ratio, %	HIIT	48.4 ± 1.4	50.2 ± 1.6	3.7	0.132
	MICT	49.1 ± 1.5	49.5 ± 1.4	0.8	0.205
	*p*-values	0.299	0.306		

Abbreviations: HIIT, high-intensity interval training; MICT, moderate-intensity continuous training.

## Data Availability

The data are not publicly available due to privacy or ethical reasons.
